# Compliance to genomic test recommendations to guide adjuvant chemotherapy decision‐making in the case of hormone receptor‐positive, human epidermal growth factor receptor 2‐negative breast cancer, in real‐life settings

**DOI:** 10.1002/cam4.6315

**Published:** 2023-07-06

**Authors:** D. Hequet, N. Hajjaji, E. Charafe‐Jauffret, A. Boucrauta, F. Dalenc, V. Nicolai, J. Lopez, O. Tredan, E. Deluche, V. Fermeaux, L. Tixier, A. Cayre, E. Menet, F. Lerebours, R. Rouzier

**Affiliations:** ^1^ Surgery Department Institut Curie St. Cloud France; ^2^ PSL St. Cloud U900 INSERM France; ^3^ Breast Cancer Department Oscar Lambret Cancer Center Lille France; ^4^ Laboratoire Protéomique, Réponse inflammatoire et Spectométrie de Masse (PRISM) University of Lille Lille U1192 Inserm France; ^5^ Department of Biopathology Institut Paoli Calmettes Marseille France; ^6^ Department of Medical Oncology Institut universitaire du cancer‐oncopole, Institut Claudius‐Regaud Toulouse France; ^7^ Department of Biopathology Hospices Civiles de Lyon Lyon France; ^8^ Department of Medical Oncology Centre Leon Berard Lyon France; ^9^ Centre de Recherche en Cancerologie de Lyon Lyon UMR5286 CNRS France; ^10^ Department of Medical Oncology CHU Limoges France; ^11^ Department of Biopathology CHU Limoges France; ^12^ Department of Biopathology Center Jean Perrin Clermont Ferrand France; ^13^ University Clermont Auvergne Clermont‐Ferrand U1240 INSERM France; ^14^ Pathology Department Institut Curie St. Cloud France; ^15^ Oncology Department Institut Curie St. Cloud France; ^16^ Surgery Department Centre François Baclesse Caen France

**Keywords:** breast cancer, deescalation, genomic tests, guidelines

## Abstract

**Background:**

Genomic tests are a useful tool for adjuvant chemotherapy decision‐making in the case of hormone receptor‐positive (HR+), and human epidermal growth factor receptor 2‐negative (HER2−) breast cancer with intermediate prognostic factors. Real‐life data on the use of tests can help identify the target population for testing.

**Methods:**

French multicentric study (8 centers) including patients, all candidates for adjuvant chemotherapy for HR‐positive, HER2‐negative early breast cancer. We describe the percentage of tests performed outside recommendations, according to the year of testing. We calculated a ratio defined as the number of tests required to avoid chemotherapy for one patient, and according to patient and cancer characteristics. We then performed a cost‐saving analysis using medical cost data over a period of 1 year from diagnosis, calculated from a previous study. Finally, we calculated the threshold of the ratio (number of tests required to avoid chemotherapy for one patient) below which the use of genomic tests was cost‐saving.

**Results:**

A total of 2331 patients underwent a Prosigna test. The ratio (performed test/avoided chemotherapy) was 2.8 [95% CI: 2.7–2.9] in the whole population. In the group following recommendations for test indication, the ratio was 2.3 [95% CI: 2.2–2.4]. In the case of non‐abidance by recommendations, the ratio was 3 [95% CI: 2.8–3.2]. Chemotherapy was avoided in 841 patients (36%) following the results of the Prosigna test. The direct medical costs saved over 1 year of care were 3,878,798€ and 1,718,472€ in the group of patients following test recommendations. We calculated that the ratio (performed test/avoided chemotherapy) needed to be under 6.9 for testing to prove cost‐saving.

**Conclusion:**

The use of genomic testing proved cost‐saving in this large multicentric real‐life analysis, even in certain cases when the test was performed outside recommendations.

## 1 INTRODUCTION

Genomic tests are part of the diagnostic spectrum to guide the indication for adjuvant chemotherapy in hormone receptor‐positive (HR+), human epidermal growth factor receptor 2‐negative (HER−) breast cancer (BC) patients. These multigene signatures allow an evaluation of the risk of recurrence and are a decision‐making support tool in intermediate‐risk situations.^1^, ^2^, ^3^ Most of the international guidelines integrate genomic tests into the decision‐making process of adjuvant treatment.^4^, ^5^ Whereas all international recommendations agree to use genomic tests in intermediate‐risk settings, the consensus on the definition of intermediate risk is subject to debate. According to NCCN (National Comprehensive Cancer Network) guidelines, the use of a gene expression assay can be considered for pT1 to pT3 tumors, even in the case of axillary lymph node involvement.^4^ ESMO guidelines include even broader indications for the use of genomic testing, without detailing biological and pathological settings.^5^ In France, genomic tests have been partially reimbursed by the national health insurance fund since 2016. In early 2019, the French Health Authority (Haute Autorité de Santé = HAS) supervised the reimbursement of these tests by conditioning their indications, limited to the case of intermediate risk of recurrence defined as pT1c‐pT2 pN0‐pN1mi grade 2 BC.^6^ The objective of controlling the indications for the prescription of genomic tests is a question of economics and cost control. However, in real‐life settings, certain tests are prescribed before and outside these indications. Moreover, very few cost‐related data are available regarding the use of genomic tests, especially within the health funding system in France, making this a topic of interest in the context of health policy and economics.^7^, ^8^, ^9^, ^10^ The aim of this study was, first, to measure the implementation over time of HAS recommendations concerning the indication of genomic tests in early BC, in real‐life settings. The secondary objective was to identify a population outside HAS recommendations for whom performing a genomic test is cost‐saving.

## 2 METHODS

We present a retrospective French multicentric study (8 centers). We included all patients consecutively managed in one of the participating centers between January 2016 and December 2020, according to the following criteria: HR‐positive, HER2‐negative early BC patients first treated by surgery, who underwent a Prosigna test to guide adjuvant chemotherapy in routine practice—whether or not the test was indicated in recommendations; all patients were candidates for adjuvant chemotherapy for HR‐positive, HER2 negative early BC according to local guidelines. The decision on chemotherapy based on the test result was made according to the risk of distant recurrence at 10 years. The threshold depended on menopausal status: >7% for premenopausal patients and >10% for postmenopausal patients. We described the percentage of tests performed outside recommendations, according to the year of testing. To identify a candidate population for testing outside current French recommendations, we calculated a ratio defined as the number of tests required to avoid chemotherapy (according to test results) for one patient, and according to patient and cancer characteristics. We then conducted cost‐saving analysis using medical cost data over a one‐year period from diagnosis from a previous study.^11^ Direct medical (medical consultation, treatments, hospitalization, imaging…) and non‐medical costs (sick leave, transportation…) were calculated based on French national health insurance fund schedules. We assessed an additional cost of €9737 in the case of adjuvant chemotherapy. We applied this cost to each patient for whom chemotherapy was avoided after the test results. The costs saved were weighed against the costs of testing; the reimbursement of tests by the health insurance fund at the time of the study was €1849 per test. Finally, we calculated the threshold of the ratio (number of tests required to avoid chemotherapy for one patient) below which the use of genomic testing was cost‐saving. This last step allowed us to select a candidate population for the use of the Prosigna test outside recommendations. Results were expressed as proportions for categorial variables, and median and ranges for continuous variables. The Chi‐square test and Fisher's exact test were used for categorical variables and Student's *t*‐test for continuous variables. A *p*‐value <0.05 was considered statistically significant. Finally, positive and negative predictive values of several combinations of imaging staging were assessed. All statistical analyses were performed using R software version 2.15.3 (http://lib.stat.cmu.edu/R/CRAN/). This study was validated by the Institutional Research Committee (N° DATA200259) of the Institut Curie, involving the project's ethical assessment. The non‐objection of each patient to the use of health data has been verified.

## 3 RESULTS

A total of 2331 patients underwent a Prosigna test during the study period. The mean age of the population was 57 years (26–90). The mean histological tumor size was 18 mm (1–71), with a mean Ki67 of 21%. The majority of patients did not present with lymph node involvement (*n* = 1612, 69%). Results of the Prosigna test are presented in Table 1.

**TABLE 1:** Results of the Prosigna test (*n* = 2331).

	*N*/median	%/range
Subtype—Prosigna		
Luminal A	1254	54%
Luminal B	1077	46%
ROR score	55	(1–94)
10y risk of distant metastasis	14	(2–51)
Risk categories		
Low	575	25%
Intermediate	929	40%
High	827	35%

Sixty‐four percent of patients (*n* = 1491) had a test performed outside recommendations. This distribution of tests according to recommendations was roughly the same in all participating centers. Patients were of similar age and with slightly higher Ki67 when testing was not indicated by HAS (S1). Test results were followed in more than 96% of patients (range 88% to 99% depending on the center), independently of recommended prescription.

**FIGURE 1:**
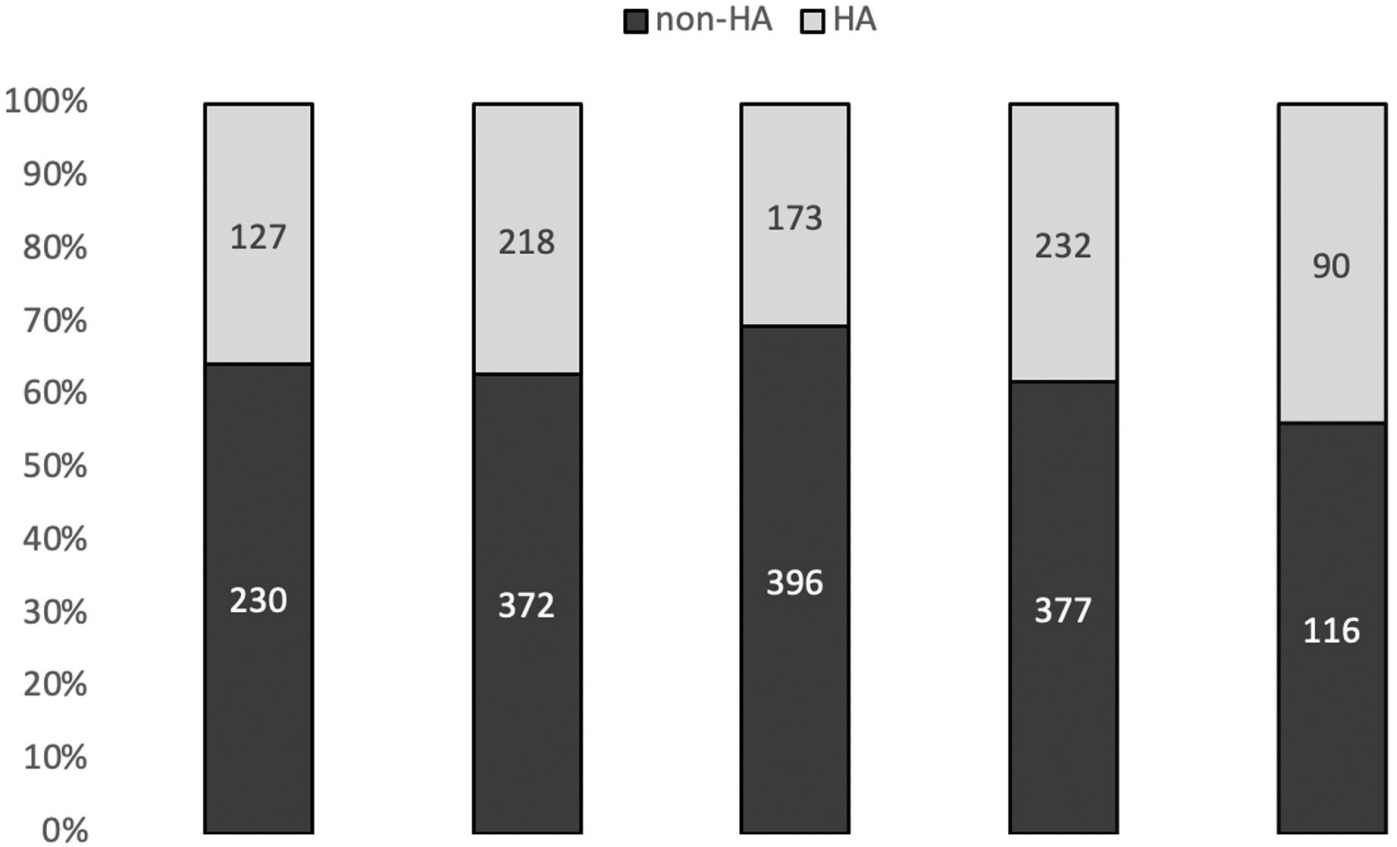
Percentage of tests performed in the HA group (within the Health Authority recommendations) and the non‐HA group (outside the Health Authority recommendations) depending on year of test.

The percentage of tests performed outside recommendations was stable from 2016 to 2019 (63%, 64%, 70%, and 62%, respectively), 2019 marking the year of HAS recommendations. In 2020, fewer tests were performed, likely due to the COVID‐19 pandemic, the percentage of tests outside recommendations dropping slightly to 56% (Figure 1).

The ratio (performed test/avoided chemotherapy) was 2.8 [95% CI: 2.7–2.9] in the full study population. In the group following recommendations for test indication, the ratio was 2.3 [95% CI: 2.2–2.4]. In the case of non‐abidance by recommendations, the ratio was 3 [95% CI: 2.8–3.2].

The ratio was within or close to the 95% CI of the “recommendations” group ratio for: pT1a and pT1b pN0 Grade 2 tumors, pT1a and pT1b pN1 Grade 1 and Grade 2 tumors, pT3 pN0 Grade 2 tumors (Table 2).

**TABLE 2:** Ratio by subgroups; in certain subgroups, the small number of patients (arbitrated at 40) was insufficient to reach conclusions (italic).

	*n*	No indication of chemotherapy*	Ratio
pT1a—pT1b			
pN0			
*Grade 1*	*21*	*17*	*1.2*
Grade 2	470	202	2.3
Grade 3	130	26	5
pN1 mic			
Grade 1	9	7	3.8
Grade 2	62	13	4.8
*Grade 3*	*8*	*0*	
pN1			
Grade 1	58	22	2.9
Grade 2	109	37	2.6
*Grade 3*	*4*	*0*	*2.9*
pT1c—pT2			
pN0			
Grade 1	57	40	1.4
Grade 2	750	324	2.3
Grade 3	75	7	10.7
pN1 mic			
*Grade 1*	*2*	*1*	*2*
Grade 2	101	17	5.9
*Grade 3*	*0*		
pN1			
Grade 1	48	9	5.3
Grade 2	207	35	5.9
*Grade 3*	*8*	*0*	
pT3			
pN0			
*Grade 1*	*6*	*4*	*1.5*
Grade 2	76	37	2.1
*Grade 3*	*20*	*8*	*2.5*
pN1 mic			
*Grade 1*	*4*	*2*	*2*
*Grade 2*	*8*	*1*	*8*
*Grade 3*	*1*	*0*	
pN1			
*Grade 1*	*15*	*9*	*1.7*
*Grade 2*	*36*	*10*	*3.6*
*Grade 3*	*1*	*0*	

* Chemotherapy indicated by the test result.

With a 36‐month median follow‐up (3–110), data on chemotherapy receipt status was available for the full population. Chemotherapy was avoided in 841 patients (36%) following the results of the Prosigna test. Cost‐saving analysis was in favor of testing. The direct medical costs saved over 1 year of care were €3,878,798 for the full population of the study and €1,718,472 in the group of patients following test recommendations.

**FIGURE 2:**
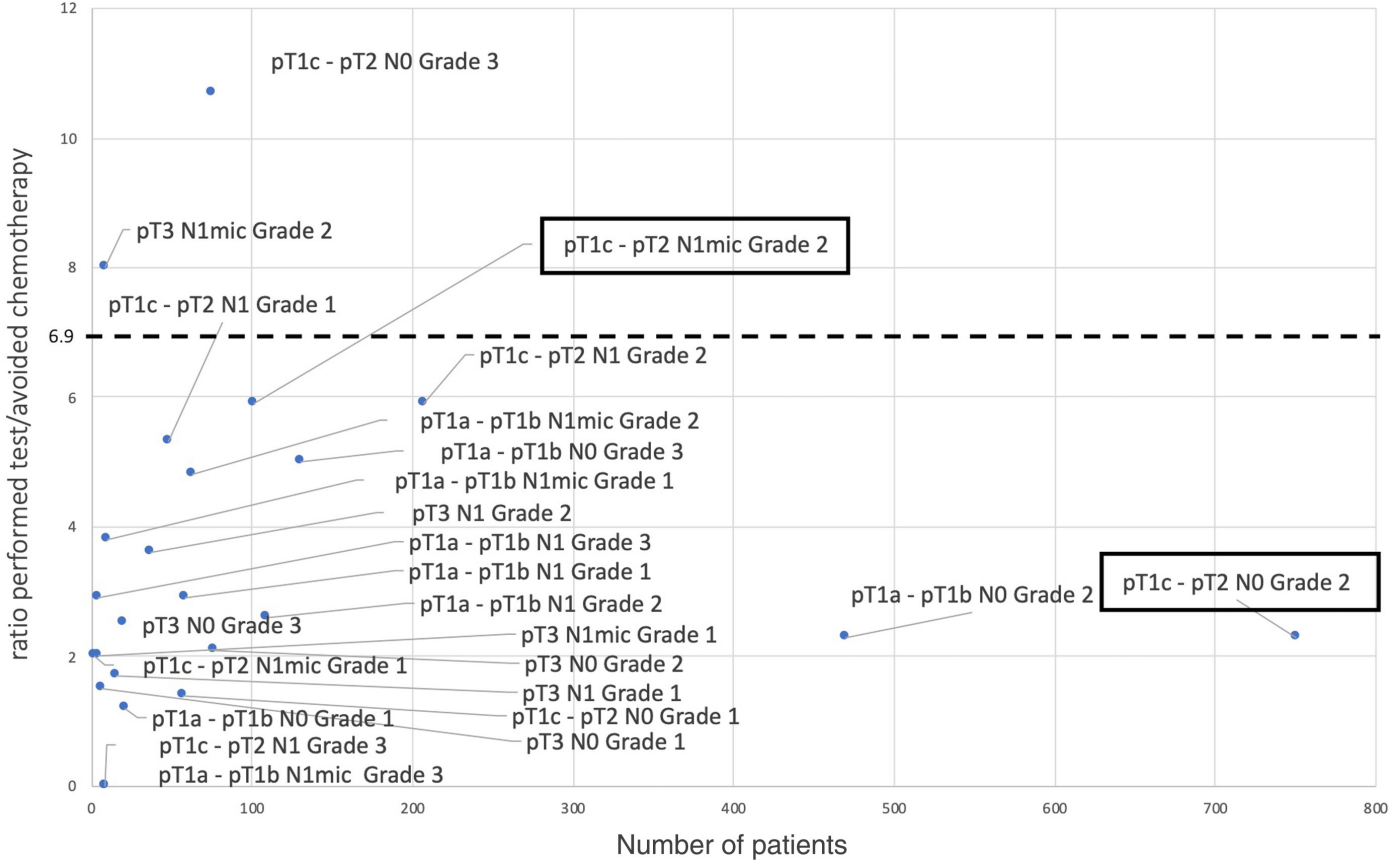
Ratio (performed test/avoided chemotherapy) for different subpopulations of the study, based on the number of patients. The framed subpopulations correspond to test indications as per recommendations. The dotted line represents the threshold of the cost‐saving analysis.

We calculated that the ratio (performed test/avoided chemotherapy) needed to be under 6.9 for testing to prove cost‐saving. This allowed us to highlight subpopulations of patients outside recommendations for whom the Prosigna test could be performed, for example, certain grade 1 tumors or pT3 tumors (Figure 2).

## 4 DISCUSSION

This large‐scale, real‐life, multicenter study demonstrates that, during the study period, more than half of Prosigna tests were performed outside of French HAS (French Health Authority) recommendations. It is important to specify that the HAS did not issue recommendations in the strict sense of the term (opposable guidelines), but rather a framework for indications of genomic testing for which reimbursement is temporary and subject to evaluation. International guidelines include broader indications for genomic tests: the NCCN strongly recommends considering genomic tests for pT1b tumors in premenopausal women (pN0) and for pT1b to pN1 in postmenopausal women.^4^ ESMO guidelines recommend the use of genomic tests, without specifying in which HR+ HER2‐ BC subpopulation.^5^ It can be noted that a decrease in the proportion of tests performed outside recommendations was observed as of 2019 (62%), the year in which test prescriptions were supervised, in comparison with 2018 (70%). In 2020, this trend was confirmed with 56% of tests conducted outside recommendations. However, we underline that the decrease in the number of tests carried out in 2020 compared to previous years is very probably linked to adapted oncology practice, in line with the healthcare situation induced by the COVID‐19 pandemic.^12^ Indeed, national recommendations were published at the beginning of the COVID‐19 pandemic. It will be interesting to continue to study the evolution of test indications in the years to come. In addition, we must emphasize that we did not include patients treated in the same centers, during the same period, who did not undergo genomic testing; whether or not a test was indicated. This could partly distort our results, particularly in terms of cost—we do not know precisely the proportion of patients who could be candidates for a test in the event of an extension of indications.

However, we can call into question the limited nature of the indications proposed by the HAS. Indeed, in certain patient subpopulations, even outside HAS recommendations, performing a genomic test was cost‐saving. In the OPTIMA Prelim trial, patients first treated by surgery with a clinically high risk of recurrence (pT >3 cm or 1 to 9 involved lymph nodes) were randomized to standard treatment (chemotherapy) or adjuvant treatment, the decision being based on genomic test results.^10^ The study reports that, in 86% of these clinical high‐risk situations, performing a genomic test (whatever available test) is cost‐saving. The direct medical and non‐medical costs of adjuvant chemotherapy (taking into account molecules, hospitalizations, consultations, complications, monitoring examinations, etc.) are very high. Performance of genomic testing is therefore cost‐saving, even when we avoid only a small proportion of chemotherapy. The OPTIMA Prelim trial was conducted in the UK. Healthcare reimbursement systems in France and the UK are very different. However, in both populations, genomic tests were cost‐saving in the majority of indications, including for larger tumors, or in the case of lymph node involvement. Therefore, in our study, genomic tests appear to be a useful and cost‐saving tool in the case of pT1a‐pT1b pN0 Grade 2 tumors, pT1a‐p1b pN1 Grade 1 and Grade 2 tumors, pT3 pN0 Grade 2 tumors. The recent publication of the results of the RxPonder study endorses this observation, demonstrating the relevance of genomic testing in postmenopausal pN1 patients.^13^

## 5 CONCLUSION

Use of genomic testing was cost‐saving in this large multicentric real‐life analysis, even in certain cases when tests were performed outside recommendations. These results highlight the possibility to extend the indications of genomic tests to more intermediate‐risk situations of HR+, HER2‐ breast cancers.

## AUTHOR CONTRIBUTIONS

**D. Hequet:** Conceptualization (lead); data curation (equal); formal analysis (equal); funding acquisition (lead); methodology (lead); project administration (lead); supervision (equal); validation (equal); writing – original draft (equal); writing – review and editing (equal). **N. Hajjaji:** Data curation (equal); formal analysis (equal); investigation (equal); writing – original draft (equal); writing – review and editing (equal). **E. Charafe‐Jauffret:** Data curation (equal); investigation (equal); validation (equal); writing – original draft (equal); writing – review and editing (equal). **A. Boucrauta:** Data curation (equal); investigation (equal); validation (equal); writing – original draft (equal); writing – review and editing (equal). **F. DALENC:** Data curation (equal); investigation (equal); validation (equal); writing – original draft (equal); writing – review and editing (equal). **V. Nicolai:** Data curation (equal); investigation (equal); writing – original draft (equal); writing – review and editing (equal). **J. Lopez:** Data curation (equal); investigation (equal); writing – original draft (equal); writing – review and editing (equal). **O. Tredan:** Data curation (equal); investigation (equal); writing – original draft (equal); writing – review and editing (equal). **E. Deluche:** Data curation (equal); investigation (equal); writing – original draft (equal); writing – review and editing (equal). **V. Fermeaux:** Data curation (equal); investigation (equal); writing – original draft (equal); writing – review and editing (equal). **L. Tixier:** Data curation (equal); investigation (equal); writing – original draft (equal); writing – review and editing (equal). **A. Cayre:** Data curation (equal); investigation (equal); writing – original draft (equal); writing – review and editing (equal). **E. Menet:** Data curation (equal); methodology (equal); writing – original draft (equal); writing – review and editing (equal). **F. Lerebours:** Investigation (equal); validation (equal); writing – original draft (equal); writing – review and editing (equal). **R. Rouzier:** Conceptualization (equal); formal analysis (equal); funding acquisition (equal); investigation (equal); methodology (equal); supervision (equal); validation (equal); writing – original draft (equal); writing – review and editing (equal).

## Supporting information


Supplementary data S1.

Data S1.

## Data Availability

On request to the corresponding author.

## References

[1] Puppe J , Seifert T , Eichler C , Pilch H , Mallmann P , Malter W . Genomic signatures in luminal breast cancer. Breast Care (Basel). 2020;15(4):355‐365. 32982645 10.1159/000509846 PMC7490652

[2] Cardoso F , van't Veer LJ , Bogaerts J , et al. 70‐gene signature as an aid to treatment decisions in early‐stage breast cancer. N Engl J Med. 2016;375(8):717‐729. 27557300 10.1056/NEJMoa1602253

[3] McVeigh TP , Kerin MJ . Clinical use of the oncotype DX genomic test to guide treatment decisions for patients with invasive breast cancer. Breast Cancer (Dove Med Press). 2017;29(9):393‐400. 10.2147/BCTT.S109847 PMC5459968 28615971

[4] NCCN Guidelines Version 4 . Invasive Breast Cancer. 2022 Accessed Februeary 11, 2022. https://www.nccn.org/professionals/physician_gls/pdf/breast.pdf

[5] Cardoso F , Kyriakides S , Ohno S , et al. Early breast cancer: ESMO clinical practice guidelines. Ann Oncol. 2019;30:1194‐1220. 31161190 10.1093/annonc/mdz173

[6] Haute Autorité de Santé . Utilité Clinique Des Signatures génomiques Dans le Cancer du Sein de Stade précoce. 2019. Accessed June 21, 2021. https://www.has‐sante.fr/upload/docs/application/pdf/2019‐01/rapport_signatures_genomiques.pdf

[7] Hequet D , Harrissart G , Krief D , et al. Prosigna test in breast cancer: real‐life experience. Breast Cancer Res Treat. 2021;188(1):141‐147. 33860387 10.1007/s10549-021-06191-x

[8] Crolley VE , Marashi H , Rawther S , et al. The impact of oncotype DX breast cancer assay results on clinical practice: a UK experience. Breast Cancer Res Treat. 2020;180(3):809‐817. doi:10.1007/s10549-020-05578-6 32170635 PMC7103011

[9] Rizki H , Hillyar C , Abbassi O , Miles‐Dua SC . The utility of oncotype DX for adjuvant chemotherapy treatment decisions in estrogen receptor‐positive, human epidermal growth factor receptor 2‐negative, node‐negative breast cancer. Cureus. 2020;12(3):e7269. doi:10.7759/cureus.7269 32195072 PMC7075474

[10] Hall PS , Smith A , Hulme C , et al. Value of information analysis of multiparameter tests for chemotherapy in early breast cancer: the OPTIMA prelim trial. Value Health. 2017;20(10):1311‐1318. 29241890 10.1016/j.jval.2017.04.021

[11] Héquet D , Huchon C , Soilly AL , et al. Direct medical and non‐medical costs of a one‐year care pathway for early operable breast cancer: results of a French multicenter prospective study. PLoS One. 2019;14(7):e0210917. 31291250 10.1371/journal.pone.0210917 PMC6619952

[12] Gligorov J , Bachelot T , Pierga JY , et al. COVID‐19 and people followed for breast cancer: French guidelines for clinical practice of Nice‐St Paul de Vence, in collaboration with the Collège Nationale des Gynécologues et Obstétriciens Français (CNGOF), the Société d'Imagerie de la femme (SIFEM), the Société Française de Chirurgie Oncologique (SFCO), the Société Française de Sénologie et Pathologie Mammaire (SFSPM) and the French breast cancer intergroup‐UNICANCER (UCBG). Bull Cancer. 2020;107(5):528‐537. 32278467 10.1016/j.bulcan.2020.03.008 PMC7118684

[13] Kalinsky K , Barlow WE , Meric‐Bernstam F , et al. 21‐gene assay to inform chemotherapy benefit in node‐positive breast cancer. N Engl J Med. 2021;385:2336‐2347. 34914339 10.1056/NEJMoa2108873 PMC9096864

