# Supplementary material for: Compliance to genomic test recommendations to guide adjuvant chemotherapy decision‐making in the case of hormone receptor‐positive, human epidermal growth factor receptor 2‐negative breast cancer, in real‐life settings
Source: Cancer Med. 2023 Jul 6;12(16):16889–95. doi: 10.1002/cam4.6315 (PMC10501273; doi:10.1002/cam4.6315)
Supplement: Supplementary file 1 — Supplementary data S1. [file CAM4-12-16889-s001.docx]

Supplementary data

S1: Patient characteristics according to HAS test indication

s
